# Continuidad de vínculos, hombres y mujeres en duelo por un ser querido: un análisis secundario*

**DOI:** 10.15649/cuidarte.3039

**Published:** 2023-12-21

**Authors:** Daniel Martínez-Esquivel, Derby Muñoz-Rojas, Pedro Ruymán Brito-Brito, Martín Rodríguez-Álvaro, Alfonso Miguel García-Hernández

**Affiliations:** 1 . Universidad de La Laguna, San Cristóbal de La Laguna, España. E-mail: alu0101456164@ull.edu.es Universidad de la Laguna Universidad de La Laguna San Cristóbal de La Laguna Spain alu0101456164@ull.edu.es; 2 . Universidad de Costa Rica, San José, Costa Rica. E-mail: derby.munoz@ucr.ac.cr Universidad de Costa Rica Universidad de Costa Rica San José Costa Rica derby.munoz@ucr.ac.cr; 3 . Universidad de La Laguna, San Cristóbal de La Laguna, España. E-mail: pbritobr@ull.edu.es Universidad de la Laguna Universidad de La Laguna San Cristóbal de La Laguna Spain pbritobr@ull.edu.es; 4 . Universidad de La Laguna, San Cristóbal de La Laguna, España. E-mail: martin.rodriguezalvaro@gmail.com Universidad de la Laguna Universidad de La Laguna San Cristóbal de La Laguna Spain martin.rodriguezalvaro@gmail.com; 5 . Universidad de La Laguna, San Cristóbal de La Laguna, España. E-mail: almigar@ull.edu.es Universidad de la Laguna Universidad de La Laguna San Cristóbal de La Laguna Spain almigar@ull.edu.es

**Keywords:** Aflicción, Enfermería, Percepción de Cercanía, Pesar, Salud Mental, Bereavement, Nursing, Perceptual Closure, Grief, Mental Health, Luto, Enfermagem, Fechamento Perceptivo, Pesar, Saúde Mental

## Abstract

**Introducción:**

El duelo es una respuesta compleja ante la pérdida de un ser querido que exhibe diferentes rutas para su ajuste, la continuidad de vínculos forma parte de su naturaleza.

**Objetivo:**

Analizar la experiencia de duelo por un ser querido en hombres y mujeres relacionada a percepción de cercanía con la persona fallecida, continuidad de vínculos y diagnósticos de Enfermería.

**Materiales y Métodos:**

Análisis secundario. Muestra a conveniencia de 251 dolientes, adultos, residentes de Canarias, hispanohablantes. Recolección con encuesta en línea compuesta por características sociodemográficas y de salud, y relacionadas con la pérdida, Escala de inclusión del otro en el yo, Escala de Continuidad de Vínculos y diagnósticos de Enfermería. Se utilizó análisis descriptivo, U de Mann-Whitney, coeficiente de Spearman. Nivel de significancia p<0,05.

**Resultados:**

Edad media de 45,09 años ±10,38. Un 22,70% (57) fue hombre, 77,30% (194) mujer. Se identificaron diferencias significativas entre hombres y mujeres en percepción de cercanía con el fallecido (p<0,05), y relaciones significativas entre percepción de cercanía con el fallecido, continuidad de vínculos y diagnósticos de Enfermería (p=0,001).

**Discusión:**

Al confrontar los resultados con otros estudios se presentan algunas consistencias y diferencias en el comportamiento de las variables demostrando el dinamismo del fenómeno.

**Conclusiones:**

Para este grupo de participantes, la experiencia de duelo no estaría ligada a construcciones sociales de género si no que contesta a una respuesta de afrontamiento según sus necesidades. La comprensión del proceso de duelo permite a la Enfermería de Salud Mental implementar acciones fundamentadas en el Proceso de Enfermería.

## Abstract (en)

**Introduction:**

Grief is a complex response to the loss of a loved one with different ways of adjustment, and Continuing Bonds are part of its nature.

**Objective:**

To analyze men's and women's experiences of grief in terms of perception of closeness to the deceased, Continuing Bonds, and Nursing diagnoses.

**Materials and Methods:**

Secondary analysis. A convenience sample of 251 Spanish-speaking adult mourners, residents of the Canary Islands, was used. Data was collected via an online survey consisting of socio-demographic, health, and loss-related characteristics, the Inclusion of Other in the Self scale, the Continuing Bonds Scale, and Nursing diagnoses. Descriptive analysis, Mann-Whitney U test, and Spearman's coefficient were used. Level of significance p<0.05.

**Results:**

The mean age was 45.09 years ±10.38 years; 22.70% (57) were male, and 77.30% (194) were female. Significant differences were found between men and women in the perception of closeness to the deceased (p<0.05), and significant relationships were found between the perception of closeness to the deceased, Continuing Bonds, and Nursing diagnoses (p=0.001).

**Discussion:**

A comparison of the results with other studies shows some consistencies and differences in the behavior of the variables, demonstrating the dynamism of the phenomenon.

**Conclusions:**

For this group of participants, the experience of grief would not be linked to social constructions of gender but instead respond to a coping response according to their needs. Understanding the grieving process allows Mental Health Nursing to implement interventions based on the Nursing Process.

## Resumo

**Introdugáo:**

O luto é uma resposta complexa a perda de um ente querido que apresenta diferentes caminhos de ajustamento, a continuidade dos lagos faz parte da sua natureza.

**Objetivo:**

Analisar a vivencia do luto por um ente querido em homens e mulheres relacionada a percepgao de proximidade com a pessoa falecida, continuidade de vínculos e diagnósticos de Enfermagem.

**Materiais e Métodos:**

Análise secundária. Amostra para conveniencia de 251 enlutados, adultos, residentes nas Ilhas Canárias, falantes de espanhol. Colegao com inquérito online composto por características sociodemográficas e de saúde, e relacionadas a perda, Escala de inclusao do outro no eu, Escala de Continuidade de Vínculos e diagnósticos de Enfermagem. Foram utilizadas análise descritiva, U de Mann-Whitney, coeficiente de Spearman. Nivel de significancia p<0,05.

**Resultados:**

Idade média de 45,09 anos ±10,38. 22,70% (57) eram homens, 77,30% (194) eram mulheres. Foram identificadas diferengas significativas entre homens e mulheres na percepgao de proximidade com o falecido (p<0,05), e relagóes significativas entre percepgao de proximidade com o falecido, continuidade de vínculos e diagnósticos de Enfermagem (p=0,001).

**Discussao:**

Ao comparar os resultados com outros estudos, apresentam-se algumas consistencias e diferengas no comportamento das variáveis, demonstrando o dinamismo do fenómeno.

**Conclusoes:**

Para este grupo de participantes, a vivencia do luto nao estaria ligada a construgóes sociais de género, mas responde a uma resposta de enfrentamento de acordo com as suas necessidades. A compreensao do processo de luto permite a Enfermagem em Saúde Mental implementar agóes pautadas no Processo de Enfermagem.

## Introducción

El ser querido se entiende como aquella persona que simboliza cercanía en distintos momentos de la biografía individual o grupal. En consecuencia, su muerte es un hecho estresante irreversible que provoca manifestaciones intensas concomitantes al duelo en las personas que sufren la pérdida^1^. Se han destacado diferencias significativas tocantes al papel que juega el sexo en el proceso de duelo demostrando que las mujeres son más susceptibles a presentar respuestas más complejas, a pesar de ello, también se ha discutido que no existiría disimilitud en la elaboración del duelo indistintamente de ser hombre o ser mujer^2^.

En suma, se ha propuesto que las mujeres tendrían mayor capacidad para enfrentar experiencias estresantes debido a su tendencia de utilizar estrategias centradas en las emociones y poseer una comunicación más abierta. Pareciera que los hombres son menos reflexivos sobre sus fortalezas personales o la importancia de las relaciones sociales, lo que limitaría sus acciones de confrontación^3^. Empero, no se piensa que una reacción sea más adecuada que otra mientras favorezcan el afrontamiento en presencia de la aflicción.

A pesar de que el duelo normal tiene connotaciones extremadamente (inter)subjetivas que requieren de una profunda reflexión para definirlo, ha emergido una controvertida necesidad de reconocer el duelo patológico. Clínicamente, se incluyeron los diagnósticos “Trastorno de duelo prolongado” y “Trastorno de duelo complejo persistente” generando opiniones contrarias^4^.

Por un lado, se ha criticado que la connotación médica podría suprimir su sentido natural dirigiéndolo a la patologización y estigmatización de la experiencia al limitarlo en un periodo de tiempo. Por otro lado, se ha argumentado que los diagnósticos del duelo ayudan a evitar el diagnóstico erróneo proporcionando normalización en las personas dolientes al reducir la culpa en aquellas que sienten que no pueden seguir adelante^5^. Sin duda, el entendimiento sobre este fenómeno se mantiene en constante evolución.

En el caso de la Enfermería de Salud Mental (ESM), el duelo está marcado por continuidades que transitan entre lo adaptativo y lo desadaptativo lejos de la idea de enfermedad^6^. Como efecto, la organización NANDA International (NANDA-I) implementó los diagnósticos de “Disposición para mejorar el duelo”, “Duelo inadaptado” y “Riesgo de duelo inadaptado”, los cuales se adecúan a las nuevas perspectivas del duelo y reflejan el juicio clínico de los(as) enfermeros(as)^7^.

Con todo, el duelo se entiende como un proceso natural autolimitado que exhibe diferentes rutas en las que la persona doliente busca ajustarse a una nueva realidad que surge tras una pérdida significativa, real, anticipada o percibida; y que presenta una gran gama de manifestaciones que influyen en su salud^7^. Es adaptativo cuando se presentan patrones de integración; para ilustrar: integrar la pérdida, recuerdos positivos o deseos de mejorar la esperanza, que son funcionales en la vida de la persona. Es desadaptativo cuando el sufrimiento ante la pérdida no sigue las expectativas socioculturales, como: estrés excesivo, experimentación de síntomas que tuvo la persona fallecida o incremento de la morbilidad^7^.

Además, la existencia de la percepción de cercanía con el ser querido fallecido sugiere que mantener lazos después de la muerte es una manifestación natural cuyas implicaciones están determinadas por la calidad de la relación que mantenían. Al respecto se introduce el concepto de continuidad de vínculos (CV). La CV es una representación mental de la persona fallecida que se mantiene continua a través de una relación activa, inclusive después de aceptar la muerte^8^.La función de la CV es difícil de determinar ante la gran diversidad de manifestaciones, sin embargo, se ha sugerido que aquellas expresiones que facilitan la aceptación de la muerte y reconstruyen significados alrededor de esta, tienden a ser adaptativas^9^. Por demás, la CV comprendería expresiones internas y externas. La CV interna buscaría la conexión psicológica con la persona fallecida, por ejemplo, imaginar el punto de vista del fallecido para la toma de decisiones. Entretanto, la CV externa anhela la conexión física, tal es confundir el aspecto físico de otro con el fallecido o sonidos con la voz del fallecido^9^.

Así bien, investigaciones antecedentes demostraron un mayor uso de la CV interna ante la muerte de un ser querido. En ese marco, la CV interna tuvo una relación positiva a la cercanía con la persona fallecida y sería un indicador de crecimiento personal, contrariamente a la CV externa que sería un indicador de duelo complicado. Por lo tanto, la CV interna se asociaría a mejores resultados en el manejo del duelo en contraposición de la CV externa que se presentaría como un factor riesgo para recabar la incorporación de la muerte^10^^,^^11^.

Otros estudios han señalado que la CV proporciona experiencias principalmente consoladoras en las personas dolientes al fortalecer la conexión y la comunicación personal por medio del cambio personal y las actividades de homenaje. Se ha resaltado que la vivencia de la espiritualidad en la CV provoca un resultado adaptativo en el proceso de duelo^12^^,^^13^. Frente a lo precedente, se reflexiona que la CV es dinámica y variable en función de cada individuo.

No obstante, recientemente no se ha comparado el comportamiento de la CV entre hombres y mujeres, por lo que este análisis haría una contribución relevante en el debate de este tema. Es importante señalar que tal comparación ayudaría a visibilizar cuáles son las necesidades de cuidado de ambos grupos. Asimismo, se considera que la observación del proceso de duelo como CV y las variables a su alrededor debe continuar para expandir su comprensión, de manera que se mejoren las prácticas de manejo del duelo basadas en la evidencia. Es conveniente establecer asociaciones entre los diagnósticos de NANDA-I y la CV desde una perspectiva de la ESM para fortalecer el Proceso de Enfermería (PE).

Ante esto, se plantea como objetivo analizar la experiencia de duelo por un ser querido en hombres y mujeres relacionada a percepción de cercanía con la persona fallecida, continuidad de vínculos y diagnósticos de Enfermería. Se disponen como hipótesis: (H1) Existen diferencias entre hombres y mujeres sobre la percepción de cercanía con la persona fallecida y la continuidad de vínculos cuando experiencian el duelo por un ser querido; (H2) Existen asociaciones entre la percepción de cercanía con la persona fallecida y la continuidad de vínculos, entre los diagnósticos de Enfermería y la continuidad de vínculos cuando las personas experiencian el duelo por un ser querido.

## Materiales y Métodos

La presentación de este documento siguió las recomendaciones de la guía Strengthening the Reporting of Observational Studies in Epidemiology (STROBE)^14^.

Se llevó a cabo un análisis secundario de datos obtenidos de una investigación que adaptó y validó la Escala de Continuidad de Vínculos (ECoVin) al español en España durante el 2021^15^. La intención del análisis fue responder preguntas relacionadas a la percepción de cercanía con la persona fallecida, al proceso de duelo como CV y a los diagnósticos de NANDA-I que no fueron consideradas.

Dicho estudio se desarrolló en el archipiélago de Canarias, Comunidad Autónoma de España y región ultraperiférica de la Unión Europea, compuesto por 8 islas volcánicas con administración propia. La población estuvo conformada por personas españolas mayores de edad, residentes en la Comunidad Autónoma de Canarias, hispanohablantes y que experimentaron la pérdida de un ser querido sin considerar el tiempo transcurrido.

La muestra fue a conveniencia, su cálculo se realizó siguiendo la regla de una proporción de diez participantes por cada ítem del instrumento a validar^16^. Se sumó la recomendación de una muestra mayor a 200 participantes para la validación de instrumentos en el área de la salud^17^. Para este estudio, se tomaron en cuenta los datos de 251 personas dolientes (n=251). Se separó la información de quienes eran menores de 18 años y de quienes no completaron la totalidad del cuestionario.

Se utilizó una encuesta en línea elaborada en Google Forms dividida en:

- Características sociodemográficas y de salud. Distinguió variables como sexo, edad, residencia, nivel de estudios, situación laboral y estado de salud general.
- Características relacionadas con la pérdida. Se consultó parentesco con la persona fallecida, edad de la persona fallecida, tiempo desde que sucedió la muerte, causa de muerte, si la muerte fue prevista o imprevista, si era el cuidador principal de la persona fallecida, si existían conflictos por resolver, si recibió ayuda para manejar la pérdida y el nivel de comunicación habitual con los demás.
- Escala de inclusión del otro en el yo (Inclusion of Other in Self scale)^(18)^. Se refiere a un instrumento pictórico que mide la percepción de cercanía con la persona fallecida en un solo ítem: “A continuación, seleccione la imagen que mejor represente cómo era de cercana para usted la relación que mantenía con la persona fallecida”; que ilustra 7 imágenes de círculos contiguos, uno representa al yo y el otro al ser querido. Las opciones de respuesta se interpretan desde la imagen 1 “menor cercanía” hasta la imagen 7 “mayor cercanía” conforme los círculos se traslapan entre sí de acuerdo al grado de inclusión. Este instrumento se utilizó para evaluar la calidad de la antigua relación entre doliente-fallecido. Concerniente a la investigación primaria, esta escala se recodificó. Los valores de las opciones de respuesta fueron reinvertidos para evitar conflictos de interpretación en el análisis.
- ECoVin (Continuing Bonds Scale)^(10)^. Es una escala de autoinforme que evalúa la relación con un ser querido después de su muerte. En la adaptación y la validación al español presentó un excelente nivel de consistencia interna (a=0,914)^(15)^. Se compone de 16 preguntas, como por ejemplo “He pensado en que la persona fallecida ha tenido una influencia positiva sobre quien soy hoy” o “He escuchado con mis propios oídos a la persona fallecida hablándome”, que utilizan una escala de Likert con 4 posibles respuestas con una puntuación de 1 a 4 donde 1 es “para nada” y 4 “constantemente”. Las puntuaciones más altas indican mayor vínculo con el ser querido fallecido, la puntuación mínima es de 16 y la máxima de 64. Asimismo, posee dos subescalas que miden la CV interna (10 ítems), y externa (6 ítems). En este análisis se reportó un Alfa de Cronbach de 0,913 para toda la escala, a=0,899 y a=0,871 para las subescalas de CV interna y externa respectivamente.
- Diagnósticos de NANDA-I7. Cogiendo como premisa los diagnósticos “Disposición para mejorar el duelo”, “Duelo inadaptado” y “Riesgo de duelo inadaptado”, la investigación valoró la presencia de las características definitorias y de los factores de riesgo en cada participante. Para la asignación diagnóstica se consideró la presencia de al menos dos características definitorias o dos factores de riesgo.

Se envió el enlace para acceder a la encuesta en línea a potenciales participantes con una carta de presentación donde se explicó el propósito junto a las particularidades del estudio, y se solicitó la colaboración para participar.

La información fue descargada de Google Forms y depurada en una base de datos que fue exportada al programa Statistical Package for the Social Sciences versión 25 (IBM SPSS 25). Los datos reportados en la investigación primaria fueron almacenados en Mendeley Data^19^. Se realizó un análisis descriptivo correlacional por dos investigadores que incluyó distribuciones de frecuencia, medidas de tendencia central y medidas de variabilidad para determinar las características de la muestra. Para conocer la existencia de diferencias significativas para dos muestras independientes se usó la U de Mann- Whitney. Por último, para establecer correlaciones entre las variables se calculó el coeficiente de Spearman. El nivel de significancia fue p<0,05. La fiabilidad de la ECoVin fue analizada con el Alpha de Cronbach. En un análisis post hoc en el programa G.Power 3.1, se identificó una potencia estadística de 0,98. No se reportaron valores perdidos.

### Consideraciones éticas

Se respetaron todos los derechos de las personas participantes garantizando su dignidad. Cada quien tuvo acceso a un consentimiento informado para aceptar o rechazar su participación. El protocolo fue aprobado por el Comité de Ética de la Investigación y Bienestar Animal de la Universidad de La Laguna (CEIBA2021-0454).

## Resultados

En el total de participantes, la media de edad fue de 45,09 años ±10,38, el 56,90% (143) era residente de Santa Cruz de Tenerife y el 43,10% (108) era residente de Las Palmas. Entretanto, el promedio de edad para los hombres (n=57) fue de 47,70 años ±10,07, para las mujeres (n=194) fue de 44,32 años ±10,37. El 71,90% (41) de los hombres y el 72,70% (141) de las mujeres, se identificaron como trabajadores independientes. La mayoría refirió no padecer ninguna enfermedad física, 89,50% de los hombres (51) y 85,60% de las mujeres (166); tampoco enfermedad mental, 96,50% (55) y 97,40% (189) correspondientemente. Otras características sociodemográficas se pueden consultar en la Tabla 1.

**Tabla 1:** Distribución de las características sociodemográficas entre hombres y mujeres en duelo por un ser querido (n=251)

Variable	Total		Hombres		Mujeres		Valor p
	(251)		(57)		(194)		
	n*	%t	n*	%t	n*	%t	
Edad en años							0,01‡
18-30	21	8,40	2	3,50	19	9,80	
31-40	57	22,80	10	17,60	47	24,20	
41-50	102	40,50	21	36,80	81	41,80	
51-60	53	21,10	19	33,30	34	17,50	
61-70	14	5,60	4	7,00	10	5,20	
71-80	4	1,60	1	1,80	3	1,50	
Lugar de residencia							0,02‡
El Hierro	2	0,80	1	1,80	1	0,50	
La Palma	22	8,70	5	8,70	17	8,80	
Tenerife	119	47,40	35	61,40	84	43,30	
Gran Canaria	94	37,50	13	22,80	81	41,80	
Fuerteventura	6	2,40	3	5,30	3	1,50	
Lanzarote	8	3,20	0	0,00	8	4,10	
Nivel de estudios							0,07
Sin estudios	1	0,40	1	1,80	0	0,00	
Sabe leer y/o escribir	1	0,40	1	1,80	0	0,00	
Primarios	2	0,80	1	1,80	1	0,50	
Secundarios	20	8,00	3	5,20	17	8,80	
Técnicos o formación profesional	36	14,30	5	8,70	31	16,00	
Universitarios	191	76,10	46	80,70	145	74,70	

Nota: n*: frecuencias absolutas; %^t^: frecuencias relativas; ♦: p<0,05.

Relativo a todas las personas participantes, en el 47,80% (120) la muerte del ser querido fue previsible o esperada, en ese sentido, el 43,80% (110) se desempeñó como el cuidador principal. Un 79,30% (199) mencionó no tener ningún conflicto por resolver con el ser querido fallecido. Además, 66,10% (166) no requirió ayuda en el proceso de duelo y 49,80% (125) manifestó un nivel de comunicación alto con los demás.

Mientras que para el 63,20% (36) de los hombres la muerte de su ser querido fue previsible o esperada, para las mujeres fue de 43,30% (84). El 36,80% (21) de los hombres y el 45,90% (89) de las mujeres se desempeñó como cuidador principal del ser querido fallecido. Referente a conflictos por resolver en la relación persona doliente - persona fallecida, 28,00% (16) de los hombres y 18,60% (36) de las mujeres consideraron que sí o que tal vez. Con respecto a si necesitaron ayuda en el proceso de duelo, el 19,30% de los hombres (11) opinaron que sí, en cuanto a las mujeres el 38,10% (74) respondieron afirmativamente. Sobre el nivel de comunicación con las demás personas, el 43,90% (25) de los hombres refirió un nivel moderado, el 53,60% (104) de las mujeres un nivel alto. La media de edad de las personas fallecidas fue de 58,04 años ±26,66. En la Tabla 2 se presentan otros datos relacionados con la pérdida.

**Tabla 2:** Distribución de las características relacionadas con la pérdida entre hombres y mujeres en duelo por un ser querido (n=251)

Variable	Total		Hombres		Mujeres		Valor p
	(251)		(57)		(194)		
	n*	%t	n*	%t	n*	%t	
Parentesco con la persona fallecida							0,88
Abuelo(a)	40	15,90	9	15,80	31	16,00	
Padre/madre	107	42,60	22	38,60	85	43,80	
Hermano(a)	19	7,60	5	8,80	14	7,20	
Hijo(a)	33	13,10	7	12,20	26	13,40	
Pareja	17	6,80	4	7,00	13	6,70	
Amigo(a)	12	4,80	5	8,80	7	3,60	
Otro	23	9,20	5	8,80	18	9,30	
Tiempo transcurrido desde la pérdida							0,99
3 meses o menos	14	5,60	4	7,00	10	5,20	
Entre 3 y 6 meses	8	3,20	2	3,50	6	3,10	
Entre 6 y 9 meses	6	2,40	1	1,80	5	2,60	
Entre 9 meses y 1 año	4	1,60	1	1,80	3	1,50	
Entre 1 y 2 años	38	15,10	9	15,80	29	14,90	
Entre 2 y 5 años	75	29,90	19	33,30	56	28,90	
Entre 5 y 10 años	39	15,50	7	12,30	32	16,50	
Entre 10 y 20 años	46	18,30	9	15,80	37	19,10	
Hace más de 20 años	21	8,40	5	8,70	16	8,20	
Causa de muerte							0,50
Enfermedad crónica	66	26,30	18	31,60	48	24,70	
Enfermedad aguda	55	21,90	10	17,50	45	23,20	
Cáncer	78	31,10	20	35,10	58	29,90	
Pérdida perinatal o durante el embarazo	14	5,60	1	1,80	13	6,70	
Accidente	22	8,70	6	10,50	16	8,20	
Suicidio	13	5,20	2	3,50	11	5,70	
Homicidio	3	1,20	0	0,00	3	1,60	

Nota: n*: frecuencias absolutas; %^t^: frecuencias relativas.

A propósito de la inclusión del otro en el yo, el total de participantes presentó una puntuación media de 5,20 ±1,91 (IC95%=4,96-5,43) lo que sugiere una cercanía considerable con la persona fallecida. En relación a la CV, se reportó una media total de 32,50 ±9,05 (IC95%=31,37-33,62) evidenciando que a menudo hay un vínculo con el ser querido fallecido.

Conexo a las subescalas, globalmente en la CV interna se presentó una media de 24,24 ±6,84 (IC95%=23,39-25,09), y en la CV externa una media de 8,25 ±3,20 (IC95%=7,86-8,65). Lo anterior significaría que el lazo interno con la persona fallecida se da a menudo y el externo se da a veces, principalmente. En la Tabla 3 se presenta la comparación de los resultados entre hombres y mujeres en duelo por un ser querido.

**Tabla 3:** Comparación de acuerdo a la percepción de cercanía con la persona fallecida, la continuidad de vínculos entre hombres y mujeres en duelo por un ser querido (n=251)

	Hombres (n=57)		Mujeres (n=194)		Valor p
	X* ± DEt	IC95%£	X* ± DEt	IC95%£	
Percepción de cercanía con la persona fallecida^§^	4,84	4,34-5,35	5,30	5,03-5,57	0,02
	±1,89		±1,91		
Continuidad de vínculos	30,91	28,65-33,17	32,96	31,67-34,26	0,09
	±8,51		±9,17		
Continuidad de vínculos internos	22,91	21,22-24,60	24,63	23,65-25,62	0,08
	±6,35		±6,95		
Continuidad de vínculos externos	8,00	7,21-8,79	8,33	7,87-8,79	0,45
	±2,98		±3,26		

Nota: X*: media; DE^t^: desviación estándar; IC95%*: intervalo de confianza 95%; §Percepción de cercanía con la persona fallecida: se midió con la Escala de inclusión del otro en el yo.Ante lo expuesto, se reportan diferencias significativas según percepción de cercanía con la persona fallecida siendo que las mujeres presentan mayor percepción que los hombres. Por lo tanto, se aceptaría parcialmente la hipótesis sobre las diferencias entre ambos grupos.

Se evidenció una correlación positiva significativa (rs=0,404; p=0,001), expresando que, a mayor percepción de cercanía con la persona fallecida, mayor es la CV. Otras correlaciones identificadas entre las variables se pueden observar en la Tabla 4.

**Tabla 4:** Correlación entre la percepción de cercanía con la persona fallecida con la continuidad de vínculos interna y externa (n=251)

		1	2	3
1	Percepción de cercanía con la persona fallecida*	1		
2	Continuidad de vínculos interna	0,396t	1	
3	Continuidad de vínculos externa	0,248t	0,533t	1

Nota: *Percepción de cercanía con la persona fallecida: se midió con la escala de inclusión del otro en el yo; ^t^: p<0,01 (bilateral). Coeficiente de Correlación de Spearman

Para finalizar, el 64,50% (162) fue diagnosticado con “disposición para mejorar el duelo”, en tanto que el 55,40% (139) con “duelo inadaptado”. La distribución por sexo para “disposición para mejorar el duelo” fue de 54,40% (31) en hombres y 67,50% (131) en mujeres, para “duelo inadaptado” fue de 36,80% (21) en hombres y de 60,80% (118) en mujeres. Sólo 1 hombre (1,80%) fue diagnosticado con “riesgo de duelo inadaptado”. Se identificaron correlaciones significativas (p=0,001) entre “disposición para mejorar el duelo” (rs=0,383), “duelo inadaptado” (rs=0,444), y “riesgo de duelo inadaptado” (rs=0,405) con la CV. A este respecto, se acepta la hipótesis sobre las asociaciones entre las variables.

## Discusión

Los resultados obtenidos de este análisis evidenciaron que existen diferencias entre hombres y mujeres que experiencian el duelo por un ser querido en la percepción que tienen sobre la cercanía con la persona fallecida, más no así en las expresiones de CV. También se identificaron correlaciones significativas entre la percepción de cercanía con la persona fallecida, la CV y los diagnósticos de Enfermería. Por ende, se acepta parcialmente la H1 y totalmente la H2. En el entendido de que dos personas no podrían experimentar las mismas respuestas de afrontamiento ante la pérdida, es que la amplia diversidad de los hallazgos convierten al duelo en un proceso complejo. No sería extraño que estos resultados no muestren consistencia con otros, más bien se esperaría^8^.

Las personas participantes se consideraron muy cercanas a la persona fallecida siendo una característica que podría favorecer el proceso de duelo. En referencia a la CV, aparenta ser más prominente la CV interna que la CV externa lo que predeciría resultados más positivos de ajuste siempre que esté ligado a encontrar un sentido a la pérdida desde un punto de vista personal, práctico, existencial y espiritual^20^.

Sin embargo, la diferencia en la percepción de cercanía con la persona fallecida entre hombres y mujeres podría estar mediada por cuestiones históricas que han definido los roles de género en relación a los pensamientos, los sentimientos y las conductas al ser querido. De la misma forma, la igualdad en la expresión de CV entre hombres y mujeres podría estar relacionada al contexto por lo que los factores socioculturales son claves para emitir un criterio científico a la vez que humano, se pone el caso de este análisis donde la mayoría reportó niveles adecuados de comunicación contrario a lo mostrado en otros estudios^4^^,^^21^.

A pesar de ello, la evidencia ha identificado diferencias significativas en el uso de CV entre hombres y mujeres señalando una mayor expresión en las mujeres^5^^,^^9^. Existe una tendencia a considerar a las mujeres más sentimentales y a los hombres menos meditativos que podría influir en el empleo de la CV^22^. No obstante, se delibera que la CV no es un proceso lineal que discrimina por sexo sino que se presenta como una oportunidad para resolver los problemas con la persona fallecida de acuerdo a las habilidades de afrontamiento de cada individuo.

Alusivo a las asociaciones sobre la percepción de cercanía con la persona fallecida, la CV y la CV interna y externa, se mostraron relaciones positivas significativas las cuales son consecuentes con otros resultados que demostraron que una mayor cercanía con el ser querido fallecido es predictiva de mayor CV interna, así como una correlación entre CV interna y CV externa. Aunque se ha sugerido que la CV externa es un predictor de menor cercanía^20^^,^^23^. Se mencionaría que no tiene sentido que las personas en duelo fomenten la desconexión con el ser querido, en oposición desarrollarían nuevos lazos que edifiquen una nueva identidad para quien ha muerto permitiendo su presencia continua en la vida diaria.

Tanto la CV interna como la CV externa han sido sugeridas como predictores de mayor intensidad en el duelo, pero sería delicado pronosticar el oficio de la CV en el proceso de duelo ya que la vehemencia de la experiencia podría relatar la relación existente antes de la muerte, desencadenando un esfuerzo mayor por mantenerla^23^. Pese a ello, se han evidenciado correlaciones positivas de CV con duelo complicado, y de CV con crecimiento personal tanto en hombres como en mujeres^4^^,^^24^. Empero, se considera que usar etiquetas para las expresiones de CV desentendiendo las creencias y las costumbres como fuentes de significado sería inadecuado.

Para terminar, los nexos entre los diagnósticos de NANDA-I y la CV reflejarían un rompimiento con los modelos tradicionales del duelo aplicándose a nuevas perspectivas más humanas que posicionan a la ESM en un ámbito amplio de comprensión. Por esta razón, los diagnósticos de Enfermería pretenden designar una respuesta que es natural en el cotidiano de muchas personas ante un suceso vital como la muerte de un ser querido evitando un tono de morbilidad^7^.

Por consiguiente, los(as) enfermeros(as) de Salud Mental interpretan el continuum en el proceso de duelo con el fin de precisar las necesidades reales sin que exista un prejuicio sino con la intención de cuidar con la persona promoviendo su adaptación ante la pérdida. En esta línea, manifestaciones inadecuadas del duelo no excluirían a las personas dolientes de expresar su disposición de mejorarlo^15^. Además, los(as) profesionales suscitarían el abordaje centrado en la persona desempeñando un papel facilitador en la recuperación y la reivindicación en el ajuste del duelo^25^.

Por medio de la relación interpersonal, la persona relataría su historia con la habilidad de modificarla, reescribirla o editarla favoreciendo su empoderamiento. Es en la propia narrativa donde las personas dolientes encontrarían el sentido de la pérdida mediante un proceso de reconstrucción de significados el cual beneficiaría la asimilación y la elaboración de la CV^26^^,^^27^. Lo propuesto, permitiría comprender los significados desde el punto de vista de cada quien para ayudarle en sus necesidades sobre la base del cuidado.

Congruente con la idea de que el estudio del proceso de duelo como CV merece tomar en cuenta una gran cantidad de dimensiones para su comprensión, los resultados de este estudio no se pueden generalizar. Se destacan como fortalezas ser de las primeras investigaciones que compara las expresiones de CV en hombres y mujeres hispanohablantes, e indaga sobre la asociación que tienen con los diagnósticos de Enfermería. Además, participan autores enfermeros especialistas en el tema del duelo y en metodología de investigación.

Algunas limitaciones de este análisis se dirigen al diseño transversal de la investigación primaria ya que restringe la naturaleza evolutiva del duelo y no establece causalidad. Encima, el cuestionario autoadministrado en línea comprometería la precisión de las medidas y provocaría sesgo de autoselección. Aparte, no se profundiza en la perspectiva de género para el desarrollo del análisis.

Es recomendable que la ESM fundamente el cuidado de las personas dolientes en el PE apoyado en la taxonomía de NANDA-I, de forma que garantice una práctica profesional y científica. Aunado, debería incluir teorías o modelos propios que permeen indiscriminadamente en la persona cuidada con la ayuda de la interacción, para profundizar en las dimensiones de su personalidad y reconocer sus necesidades reales. Así las acciones que se implementen deben estar basadas en la evidencia siendo específicas para cada individuo o grupo.

En cuanto a la investigación, se hace necesario desarrollar estudios longitudinales que permitan observar el comportamiento de la CV y la evolución de los diagnósticos de NANDA-I durante la adaptación al duelo. Sería importante apoyar el análisis de este fenómeno en la perspectiva de género integral para adquirir mayor sensibilidad de las vivencias de las personas dolientes evitando equipararlas. En virtud de ello, se recomienda mayor representatividad de hombres u otras poblaciones.

## Conclusiones

Al menos para este grupo de participantes, la experiencia de duelo como CV no estaría ligada forzosamente a construcciones sociales de las funciones que tienen los hombres y las mujeres si no que contesta a una respuesta de afrontamiento según las necesidades de cada persona, a la vez que se relaciona a la percepción de cercanía con la persona fallecida sin que signifique un padecimiento sino una actitud de amor. A razón de los diagnósticos de Enfermería, ilustrarían una respuesta humana natural de una persona doliente que podría requerir acciones de cuidado para fortalecer su adaptación frente a la muerte.
